# P-2163. Accumulative Incidence and Outcomes of Cytomegalovirus Disease in Kidney Transplant Recipients during Pre- and Post- Protocol Revision Periods in Thammasat University Hospital

**DOI:** 10.1093/ofid/ofaf695.2326

**Published:** 2026-01-11

**Authors:** Thitima Phairoch, Sasinuch Rutjanawech

**Affiliations:** Thammasat University Hospital, HuaHin, Prachuap Khiri Khan, Thailand; Thammasat University, Khlong Luang, Pathum Thani, Thailand

## Abstract

**Background:**

Cytomegalovirus disease (CMV) remains a leading infectious complication in kidney transplant recipients (KTRs). In resource-limited settings, antiviral prophylaxis might not always be provided and routine viral load (VL) monitoring aiming for preemptive treatment is often unavailable due to financial and infrastructural constraints. We assessed the impact of implementing monthly CMV VL monitoring in intermediate-risk KTRs on CMV-related outcomes and allograft function.

**Figure 1:**
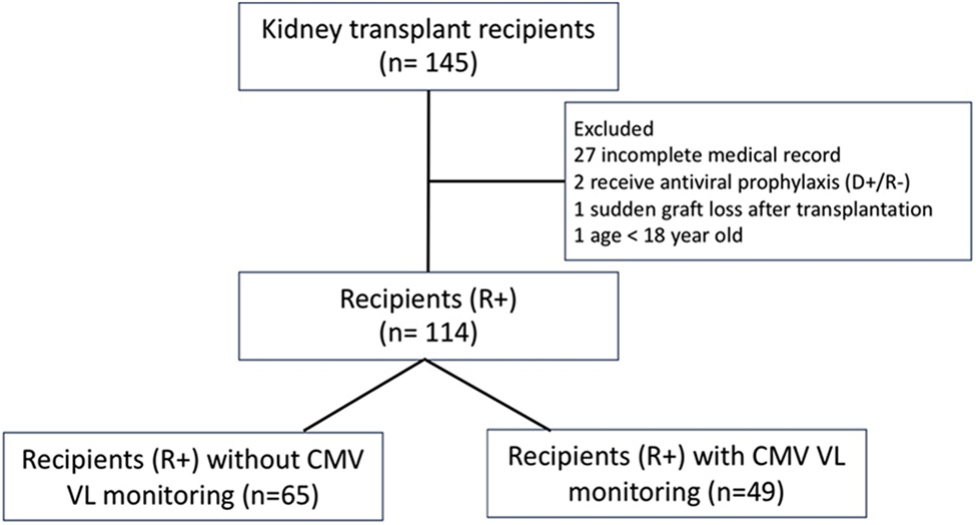

**Figure:**
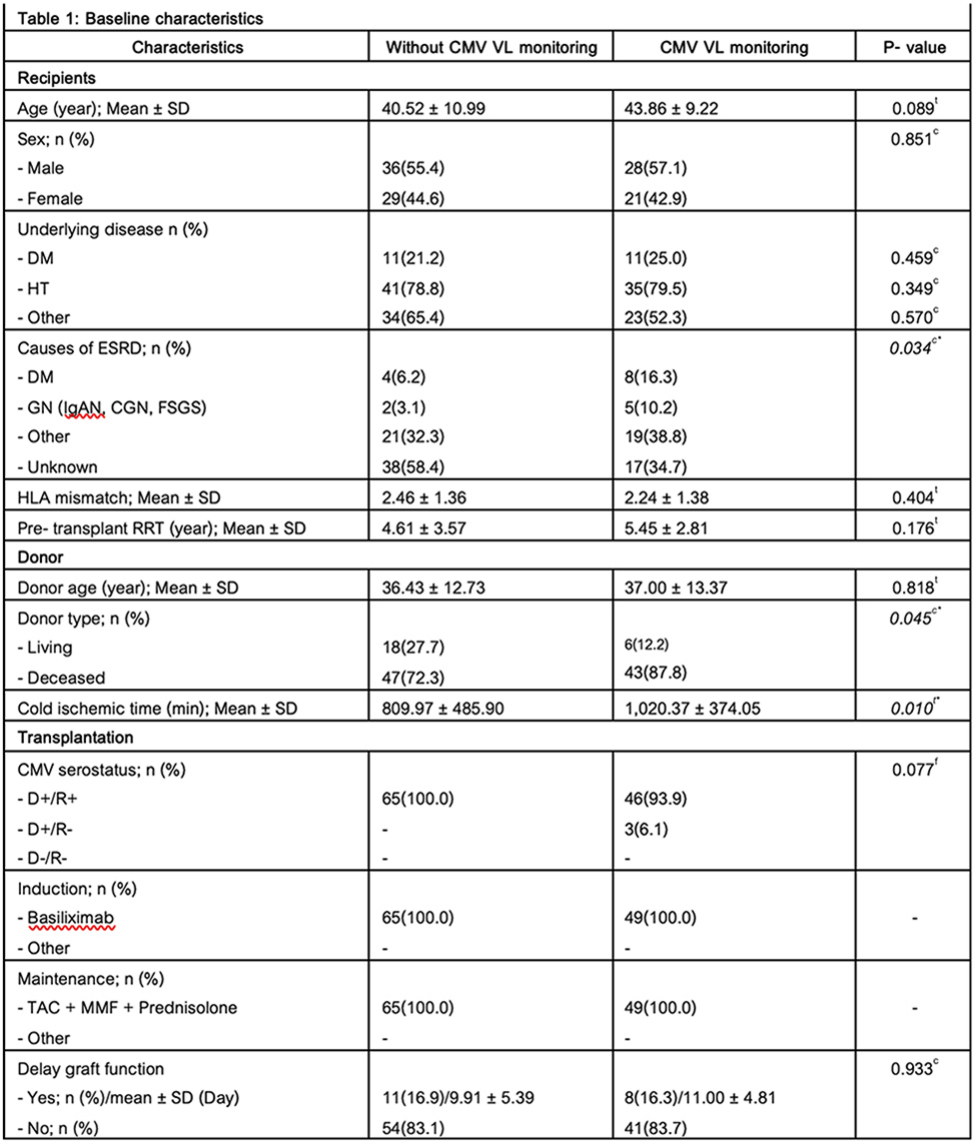
Baseline characteristics

**Methods:**

We conducted a mixed-method prospective and retrospective cohort study of adult KTRs at Thammasat University Hospital from January 2012 to September 2024. Patients were categorized into two groups: pre-protocol revision (no CMV VL monitoring) and post-revision (monthly CMV VL monitoring). Primary outcome was incidence of CMV disease; secondary outcomes included incidences of CMV syndrome and viremia, graft outcomes, and antiviral treatment duration.

**Figure:**
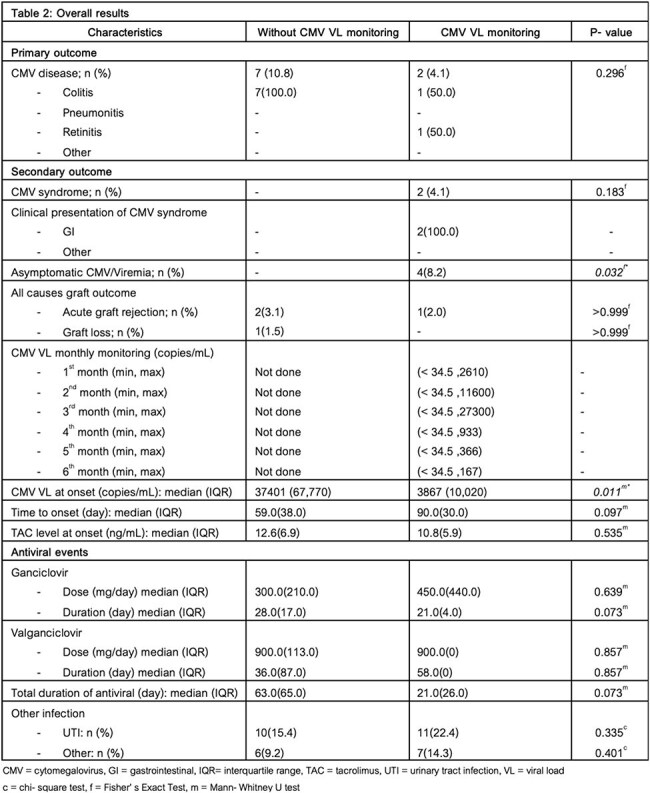
Result

**Results:**

Among 114 intermediate-risk recipients (D+/R+), 65 (57%) received no CMV VL monitoring and 49 received monthly monitoring. CMV disease occurred in 10.8% of patients without monitoring versus 4.1% with monitoring (p=0.296). Asymptomatic viremia was identified only in the monitoring group (8.2%, p=0.032), allowing for early intervention. Median CMV VL at onset was significantly lower with monitoring (3,867 vs. 37,401 copies/mL, p=0.011), and antiviral treatment duration was shorter (21 vs. 63 days, p=0.073). No significant differences were observed in acute rejection or graft loss.

**Conclusion:**

Although not statistically significant, CMV VL monitoring was associated with a lower incidence and earlier detection of CMV disease, lower viral burden, and shorter antiviral treatment. This study supports the feasibility and potential benefit of protocol-based CMV monitoring in resource-limited settings to improve post-transplant outcomes.

**Disclosures:**

All Authors: No reported disclosures

